# Using Whole-Genome Sequencing Data Reveals the Population Structure and Selection Signatures for Reproduction Traits in Duolang Sheep

**DOI:** 10.3390/ani15233466

**Published:** 2025-12-01

**Authors:** Keyao Wang, Qianjun Li, Zhigang Niu, Zhengfen Xue, Shiyuan Li, Jiabao Yan, Yang Chen, Yanlong Zhang, Hongcai Shi, Xiangdong Ding

**Affiliations:** 1 State Key Laboratory of Animal Biotech Breeding/National Engineering Laboratory for Animal Breeding, College of Animal Science and Technology, China Agricultural University, Beijing 100193, China; 18045103002@163.com (K.W.); lqj1821168324@163.com (Q.L.); li_shiyuan_2000@163.com (S.L.); 2 Key Lab of Reproduction & Breeding Biotechnology of Grass Feeding Livestock of MOA, Xinjiang Academy of Animal Science, Urumqi 830000, China; xjnzg@126.com (Z.N.); xzf780329@126.com (Z.X.); yjb980706@163.com (J.Y.); xjmlcy@126.com (Y.C.); zylong929@163.com (Y.Z.)

**Keywords:** duolang sheep, population genetic structure, selection signatures, reproductive traits

## Abstract

Duolang sheep, a meat–fat dual-purpose breed indigenous to Xinjiang, China, has been cultivated traditionally by the local Uyghur people for its prolificacy and precocious sexual maturity, while little research on the population structure and trait inheritance characteristics of Duolang sheep is available. This study employed whole-genome resequencing data from a cohort of 60 Duolang sheep to dissect their genetic population structure and genes related to reproductive traits. A total of 1565 Gb of high-quality data with an average depth of 14.06× was generated. After SNP calling and quality control, 31,300,060 SNPs were identified. Following linkage disequilibrium (LD)-based pruning, a total of 4,479,177 high-quality SNPs were retained for subsequent analyses. Based on these SNPs, the internal genetic structure of the Duolang sheep population was elucidated, with 14 kinship outliers detected through principal component analysis (PCA). Furthermore, LD decay analysis revealed that the r^2^ declined below 0.1 at approximately 10 kb, indicating a relatively low level of selection pressure in the population. Within the population, Tajima’s D and iHS methods detected 517,218 and 82,534 candidate SNPs under selection, respectively, with 24,453 SNPs overlapping between the two methods. By splitting Duolang sheep into single-lamb (*n* = 29) and multiple-lamb (*n* = 12) subgroups according to litter size, 267,654 SNPs were identified by XP-CLR, while 184,179 SNPs suffering from selection were detected by *F_ST_* and 62,150 by XP-EHH. Functional enrichment analysis of selected genes reveals the selection directions (domestication, growth, and reproduction) and related candidate genes in the Duolang sheep population, including *ESRRA*, *ESRRB*, *OXT*, *FSHR*, *ESR2*, *GNRHR*, and *BMPR1B*. This study provides the first comprehensive genomic landscape of Duolang sheep, elucidating genetic signatures of its adaptive traits.

## Simple Summary

Duolang sheep are a special local breed from Xinjiang, China, known for their high fertility and early maturity. They have been traditionally raised by the Uyghur people for both meat and fat production. However, little was known about their genetic background and the reasons behind their strong reproductive performance. In this study, we analyzed the whole-genome of 60 Duolang sheep to explore their genetic structure and identify genes related to reproduction. The results revealed rich genetic diversity and relatively low selection pressure in the population, indicating strong adaptability. We also detected several genetic regions that may be associated with reproductive traits. This research provides the first comprehensive genomic overview of Duolang sheep and offers valuable insights for conserving and improving this unique local breed, ensuring its sustainable development and continued contribution to local animal husbandry.

## 1. Introduction

China has rich resources in sheep. According to geographical distribution and genetic relationships, domestic sheep can be divided into three major lineages: Tibetan sheep, Mongolian sheep, and Kazakh sheep. Mongolian sheep are mainly distributed in high latitudes and adapted to cold climates. Kazakh sheep have a strong fat accumulation ability and can store a large amount of fat to meet the nutritional needs in winter. Tibetan sheep mostly live in low latitude areas with warm and less snowfall, and have strong adaptability. In 2023, sheep production in Xinjiang reached 37.15 million, emphasizing the important role of Xinjiang in the national supply of sheep meat and its crucial contribution to ensuring an efficient national supply of livestock products.

Reproductive performance is one of the most economically important traits in modern sheep production, as it directly influences flock productivity, lambing rate, and farm profitability. Improving reproductive efficiency through genetic selection can increase the number of lambs produced per ewe, accelerate genetic progress, and reduce production costs. However, reproductive traits are complex quantitative traits that are influenced by both genetic and environmental factors, and their molecular mechanisms remain not fully elucidated. Previous studies have shown that mutations in genes such as BMPR1B, BMP15, and GDF9 have significant effects on ovulation rate and litter size, highlighting the crucial role of genetic variation in determining reproductive performance. Understanding the genetic basis of reproduction is therefore essential for improving breeding efficiency and sustaining the productivity of sheep populations.

In recent years, research on Duolang sheep has provided valuable insights into the genetic mechanisms underlying key production traits. For instance, a whole-genome resequencing analysis identified *SFRP4* as a potential major gene affecting lumbar vertebrae count, with the potential to significantly enhance meat yield in Duolang sheep [1]. RNA-seq and bioinformatics techniques were employed to identify differentially expressed lncRNAs and genes in subcutaneous fat tissue, exploring key molecular mechanisms underlying fat deposition in Duolang sheep [2]. Furthermore, transcriptomic profiling of ovarian tissues from multi-fetal sheep has revealed several key genes, including *COL3A1*, *RPS8*, and *ACTA2* [3].

In reproduction traits, the number of lambs born per litter significantly impacts the production efficiency of the sheep farming industry. However, little research on the litter size of Duolang sheep is available. The selection signature refers to the evidence of selection detected by analyzing the non-random distribution of genomic variations within a population. Benefiting from the development of high-throughput sequencing technology, selection signatures could be more efficiently identified in the genome. Selection signatures is used to identify which genes or genomic regions are under positive selection, balancing selection, or negative selection, thereby revealing the molecular mechanisms of how organisms adapt to their environments. These methods can be divided into two categories depending on the different types of clustering information: within-population methods, such as Tajima’s D [4] and the integrated haplotype score (iHS) [5], and methods based on comparisons between two groups, e.g., the cross-population composite likelihood ratio (XP-CLR) [6], the fixation index (*F_ST_*) [7], and the cross-population extended haplotype homozygosity (XP-EHH) [8]. In addition, selection signatures can be detected based on single-point variants or haplotype expansions to further reveal the adaptive evolution of the genome. Using these methods, numerous studies have identified key genomic regions and genes suffering from natural or artificial selection in sheep. Selection signatures were detected in South African Mutton Merino sheep through whole-genome sequencing, revealing multiple gene regions associated with growth and meat quality [9]. The genomic diversity of Chinese indigenous chickens was analyzed, along with selection signatures in Chinese gamecock chickens, identifying candidate genes associated with muscular development and behavioral patterns [10]. A study on cold acclimatization in Weining cattle analyzed the genes *UBE3D* and *ZNF668*, which may be associated with fat metabolism and blood pressure regulation in response to cold environments [11].

In the current study, Tajima’s D and iHS analysis methods were applied to identify regions subject to selection or are currently under selection in Duolang sheep. Meanwhile, the XP-CLR method was employed as the primary approach to detect selection regions associated with litter size, with *F_ST_* and XP-EHH analyses applied as complementary methods to validate the identified signals in the single-lamb and multiple-lamb subpopulations. The results are expected to help understand the genetic basis underlying Duolang sheep and the molecular mechanisms relevant to reproductive traits and to provide significant genetic insights for future sheep breeding programs.

## 2. Materials and Methods

### 2.1. Animals and Phenotypic Data

A total of 60 healthy adult sheep (55 ewes and 5 rams) were selected from pastures in Xinjiang, China. Following animal ethics and welfare guidelines, all animals were sampled by collecting ear vein blood samples and used to perform whole-genome resequencing using the Illumina NovaSeq6000 PE150^TM^ platform (Illumina, San Diego, CA, USA). Among 41 ewes, 29 had a litter size of one, 11 had two, and 1 had three.

### 2.2. Variant Calling and Filtering

For the whole-genome sequencing data, Trimmomatic v0.39 was utilized to remove low-quality reads [12], followed by alignment of high-quality paired-end reads to the *Ovis aries* reference genome (oviAri4) with the BWA-MEM algorithm v0.7.17 [13]. SAMtools v1.10 was then used to generate and sort BAM files [14], and PCR duplicates were removed via Sambamba v9.5.9 [15]. The Genome Analysis Toolkit (GATK, v4.1.9) was implemented for SNP calling across all samples [16,17].

Population-level variants were subsequently merged and subjected to stringent filtering with GATK v4.1.9 and BCFtools v1.10.2 [14], excluding sites with Quality by Depth (QD) less than 2.0; Mapping Quality (MQ) less than 40.0; Fisher’s Exact Test for strand bias (FS) greater than 60.0; Strand Odds Ratio (SOR) greater than 3.0; Mapping Quality Rank Sum Test (MQRankSum) less than −12.5; Read Position Rank Sum Test (ReadPosRankSum) less than −8.0; Minor Allele Frequency (MAF) less than 0.01; call rate less than 10%; or non-biallelic SNPs.

Finally, functional annotation of filtered variants was conducted using ANNOVAR v2020.06.07 [18].

### 2.3. Heterozygosity Rate and Inbreeding Coefficients

To assess the heterozygosity rate, inbreeding coefficients (*F*_ROH_ and *F*_HOM_), polymorphism information content (PIC), and nucleotide diversity (π), PLINK v1.90 [19] and vcftools v0.1.16 [20] were used in this study. The runs of homozygosity (ROH) were identified through the --homozyg function with the following parameters: --homozyg homozyg-density 100, --homozyg-gap 100, --homozyg-snp 50, --homozyg-kb 500, --homozyg-window-het 3, --homozyg-window-missing 5, --homozyg-window-snp 50, and --homozyg-window-threshold 0.05. Nucleotide diversity (π) was calculated using VCFtools v0.1.16 with the --window-pi 10,000 option.

### 2.4. Population Genetic Structure Analysis

Based on the filtered SNPs, principal component analysis (PCA) and LD decay analysis were conducted to investigate the population structure of Duolang sheep. Prior to PCA, SNPs were pruned for LD using PLINK v1.90 with a sliding window spanning 100 SNPs and a step size of 50 SNPs, excluding SNP pairs with an r^2^ greater than 0.5. The genetic relationship matrix was subsequently constructed via gcta64 v1.93.3 [21], and principal components were derived to assess population substructure. The LD decay analysis was conducted using PopLDdecay v3.41 [22] with the following settings: SNP sites within 300 kb, a minimum allele frequency of 0.005, and the calculation of r^2^ values between two SNP sites. All plots were generated using R v4.3.2.

### 2.5. Selective Sweep Analysis

Selective sweep analyses were performed using both within-population and between-group comparisons. To identify genomic regions under selection, Tajima’s D was calculated using VCFtools v0.1.16, and the integrated haplotype score (iHS) was computed using selscan v1.3.0 [23].

Tajima’s D values were calculated in non-overlapping 50 kb sliding windows, where those corresponding to the top and bottom 1% of Tajima’s D values were considered regions under selection. The iHS test was applied to identify genomic regions under selection based on linkage disequilibrium. After normalization, the markers with the highest and lowest 0.05% of scores ($\left|iHS\right|>3$) were considered as being under selection.

For the detection of genomic regions under selection between the single-lamb (*n* = 29) and multi-lamb (*n* = 12) subgroups, the cross-population composite likelihood ratio (XP-CLR) method was used as the primary analytical approach. XP-CLR was performed using xpclr v1.1.2 [6]. Genomic windows falling within the top 1% of XP-CLR scores were identified as candidate regions under selection. To validate the selection signals detected by XP-CLR, two additional population genetic methods were applied. First, the *F_ST_* were calculated using a sliding window approach with a window size of 50 kb and a step size of 20 kb with VCFtools v0.1.16, and the top 1% of windows were retained as divergent regions. Second, prior to XP-EHH analysis, genotype data were phased using Beagle v5.4 to reconstruct haplotype structure. The phased data were then used as input for selscan v1.3.0 [23]. XP-EHH scores were normalized using the norm module, and loci falling within the top and bottom 0.05% of the distribution were considered under selection.

Regions identified by Tajima’s D, XP-CLR, and *F_ST_* were extended by ±150 kb, while loci identified by iHS and XP-EHH were expanded by ±25 kb. Gene annotation within these selected regions was performed using the BioMart database (http://asia.ensembl.org/biomart/martview, accessed on 26 November 2025).

### 2.6. Gene Set Enrichment Analysis

To investigate the functional relevance of candidate genes, Gene Ontology (GO) enrichment and the Kyoto Encyclopedia of Genes and Genomes (KEGG) pathway were performed using Database for Annotation, Visualization and Integrated Discovery (DAVID) v6.8 [24,25]. Gene sets were derived from overlapping regions identified by within-population scans (e.g., Tajima’s D, iHS) and between-population scans (e.g., XP-CLR). GO terms and KEGG pathways with a *p*-value less than 0.05 were considered statistically significant.

## 3. Results

### 3.1. Population Genetic Variation

**Figure 1:**
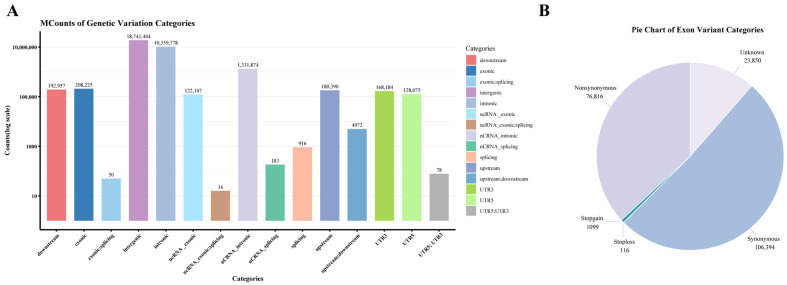
SNP annotation of Duolang sheep. (**A**) Genomic annotation of SNPs according to ANNOVAR. (**B**) Pie chart of SNPs annotated in exonic regions.

Whole-genome resequencing of 60 Duolang sheep achieved an average depth of 14.06×, generating 1565 Gb of clean data after filtering. Subsequently, 31,300,060 SNP sites were identified, with functional annotation revealing the counts and genomic regions for each SNP (Figure 1A).

The functional annotation revealed genomic distribution patterns of Duolang sheep, with 59.61% (18,741,444) localized to intergenic regions. Protein-coding regions harbored 0.66% (208,225) of variants, of which 36.9% (76,816) were nonsynonymous variants. Transcriptional regulatory elements contained 1.14% of SNPs, including 0.53% (168,184) in 3′UTRs and 0.41% (128,075) in 5′UTRs (Figure 1B). It should be noted that a single SNP may be assigned to multiple functional categories depending on overlapping transcript models (e.g., exonic and splicing, or upstream and 5′UTR). Therefore, the total number of SNPs across all categories may exceed the number of unique variant sites.

### 3.2. Genetic Diversity of the Duolang Sheep Population

Genome-wide heterozygosity averaged 0.2020 (range 0.1780–0.2210). The inbreeding coefficient *F*_ROH_ remained at 0.00023 (0.00020–0.00028), while the estimated *F*_HOM_ inbreeding coefficient was 0.01520 (−0.0500–0.1500), both indicating a low level of inbreeding and a relatively stable genetic structure. The mean PIC was 0.1639, reflecting low polymorphism at some loci. Nevertheless, the nucleotide diversity was 0.2588, indicating a relatively high level of genome-wide variation. Collectively, these findings suggest that the Duolang sheep population maintains genetic stability while preserving a moderate degree of genetic diversity, with an overall balanced genetic structure.

### 3.3. Population Structure Analysis

**Figure 2:**
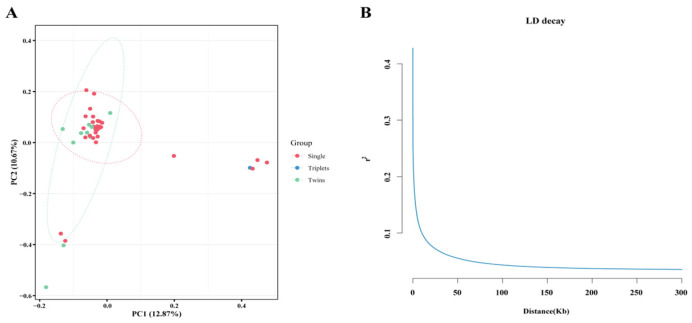
The population structure of 41 Duolang sheep. (**A**) The distribution of 41 Duolang sheep on PC1 and PC2. (**B**) The decay of r2 with distance for the 60 Duolang sheep.

Following linkage disequilibrium pruning, 4,479,177 high-quality SNPs were retained for population structure analysis. PCA revealed limited genetic stratification, with the first two principal components explaining 25.39% of the variance. Among the 55 Duolang sheep, 14 individuals showed outlier characteristics in terms of kinship compared to the entire population. Of these, two individuals were triplets, four were twins, and eight were single-lamb individuals (Figure 2A).

Linkage disequilibrium decay patterns further characterized the population’s evolutionary history, with pairwise r^2^ values declining to background levels (r^2^ < 0.1) within 10 kb physical distances (Figure 2B). This rapid LD decay suggests a historically large effective population size and minimal recent selective sweeps, consistent with the observed genetic homogeneity.

### 3.4. Selective Sweep Analysis

**Figure 3:**
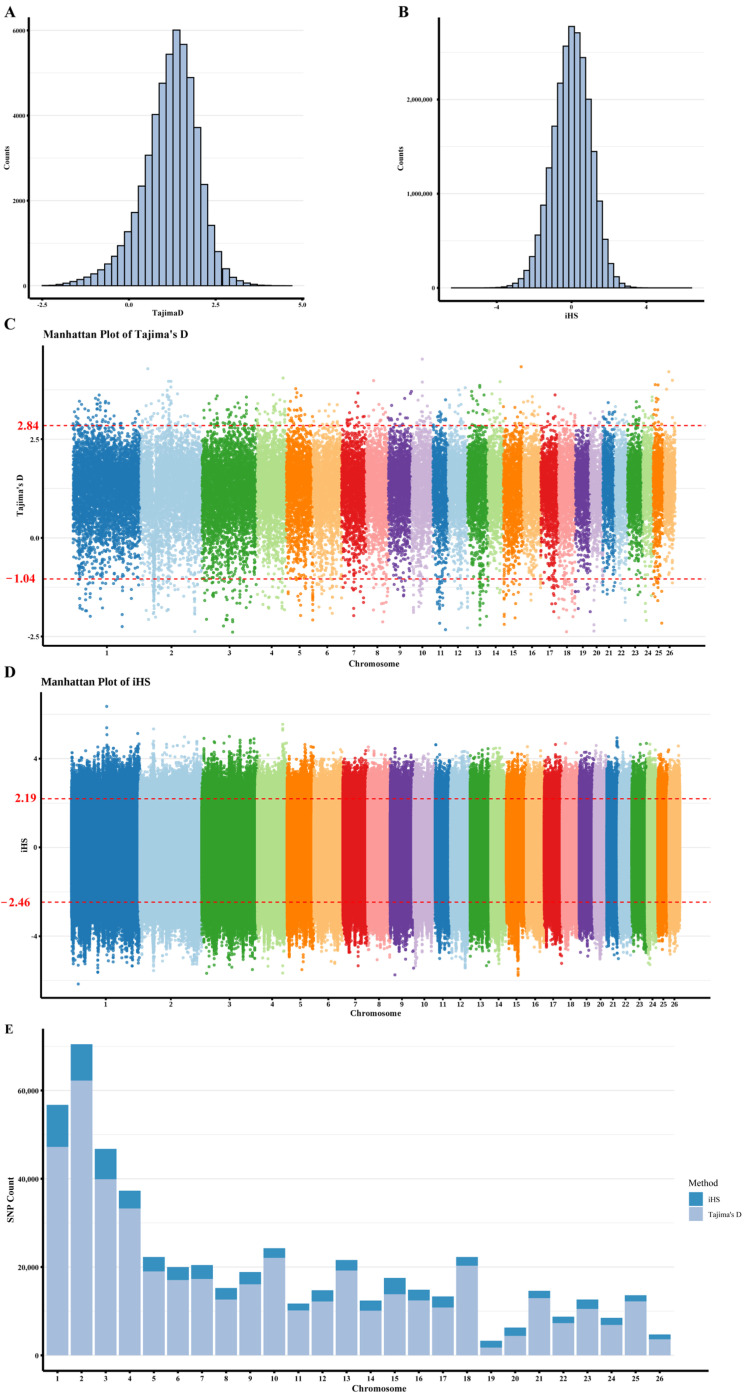
Within-population selective sweep analysis of 60 Duolang sheep. (**A**) Genome-wide distribution of Tajima’s D values. (**B**) Genome-wide distribution of iHS values. (**C**) Manhattan plot of iHS values highlighting candidate regions. (**D**) Manhattan plot of Tajima’s D values highlighting candidate regions. (**E**) Chromosomal distribution of selected SNPs identified by iHS and Tajima’s D analyses.

The distribution of Tajima’s D or iHS scores for each window or locus across the whole genome and their distribution across each chromosome is shown in Figure 3. Based on the top 1% extreme Tajima’s D scores, 1036 windows were identified (including 517,218 SNPs), with 518 windows under balancing selection and 518 windows under purifying selection. Among them, chromosomes 1, 2, 3, and X have the most selected SNPs, with 47,224, 62,249, 39,885, and 61,814 SNPs, respectively. The region suffering the most significant selection is on chromosome 10, with an average Tajima’s D statistic of 4.53. The region spans from 43,100 kb to 43,150 kb. In the 1% windows with the highest and lowest Tajima’s D scores, a total of 3670 genes were collected within a 350 kb region upstream and downstream, including genes related to reproduction such as *ESRRA* and *ESRRB*.

A total of 80,887 SNP sites were selected based on the most extreme 0.1% of iHS scores ($\left|iHS\right|>3$), among which 24,453 SNPs were within the windows detected by Tajima’s D. Among them, chromosomes 1, 2, 3, and 4 have the most selected SNPs, with 9507, 8224, 6891, and 4031 SNPs, respectively. The region under the most significant negative selection is at position 27,021.3 kb on chromosome 1, with a standardized iHS statistic of −6.16. The region under the most significant positive selection is at position 141,518.9 kb on chromosome 1, with a standardized iHS statistic of 6.36. In the regions within 25 kb upstream and downstream of the 0.1% most extreme iHS scores (|iHS| > 3), a total of 4157 genes were collected, including genes related to reproduction such as *OXT*, *FSHR*, *ESR2*, *ESRRB*, *GNRHR* and *BMPR1B*.

**Figure 4:**
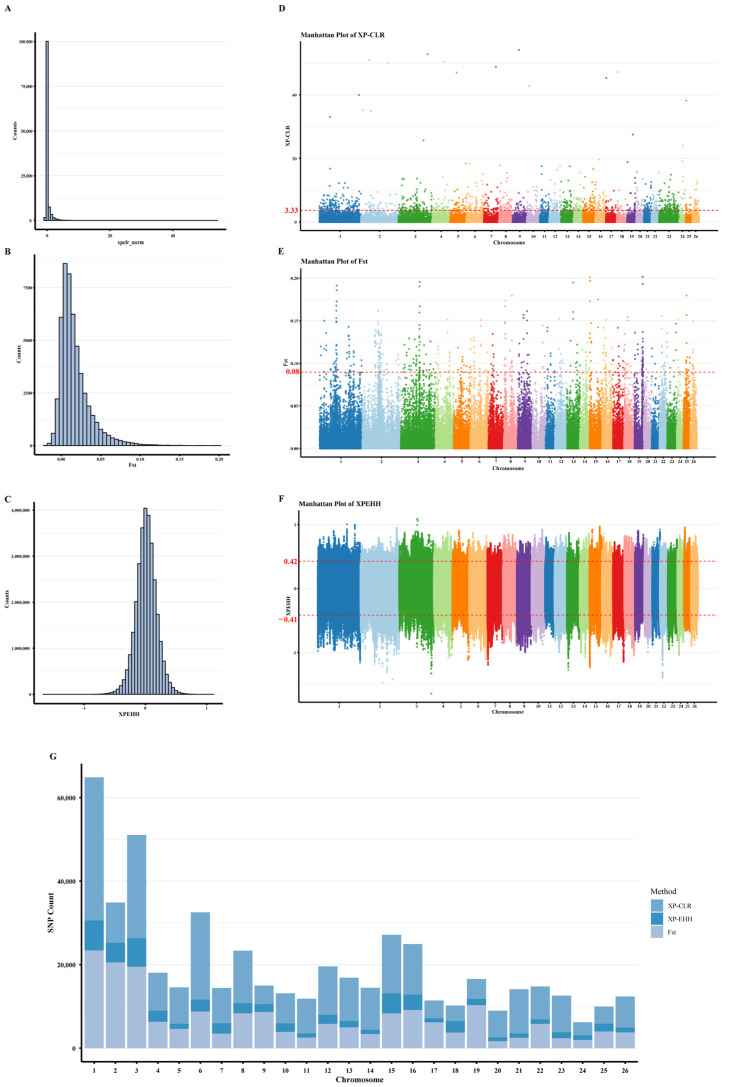
Between-population selective sweep analysis of 41 Duolang sheep. (**A**) Genome-wide distribution of XP-CLR values. (**B**) Genome-wide distribution of *F_ST_* values. (**C**) Genome-wide distribution of XP-EHH values. (**D**) Manhattan plot of XP-CLR values highlighting regions of genetic differentiation. (**E**) Manhattan plot of *F_ST_* values highlighting candidate regions. (**F**) Manhattan plot of XP-EHH values highlighting candidate regions. (**G**) Chromosomal distribution of the selected SNPs identified by XP-CLR, *F_ST_* and XP-EHH analyses.

Using XP-CLR as the primary method, a 20 kb sliding window was applied, and the top 1% of windows were retained, identifying a total of 267,654 SNPs under selection. Chromosomes 1, 2, 3, and 19 contained the largest numbers of selected SNPs, with 23,406, 20,542, 19,506, and 10,320 SNPs, respectively. Within 150 kb upstream and downstream of these top XP-CLR windows, a total of 2806 genes were collected (Figure 4). From the 50 kb windows with *F_ST_* statistics greater than 0, the top 1% windows with the highest scores were selected as the most significantly differentiated population genetic regions, totaling 480 windows (containing 184,179 SNPs). Among them, chromosomes 1, 2, 3, and 19 have the most selected regions, with 23,406, 20,542, 19,506, and 10,320 SNPs, respectively. In the 1% windows with the highest *F_ST_* scores, a total of 596 genes were collected within a 150 kb region upstream and downstream. A total of 62,150 most significantly differentiated selected SNPs were identified based on XP-EHH statistic scores. Among these, chromosomes 1, 2, 3, and 15 have the most selected regions, containing 7182, 4692, 6866, and 4790 SNPs, respectively. In the regions within 25 kb upstream and downstream of the 0.05% most extreme XP-EHH scores, a total of 1234 genes were collected, including genes related to reproduction such as *ESRRA*, *BMPR1B,* and *IGF1R* (Table 1).

**Figure A1:**
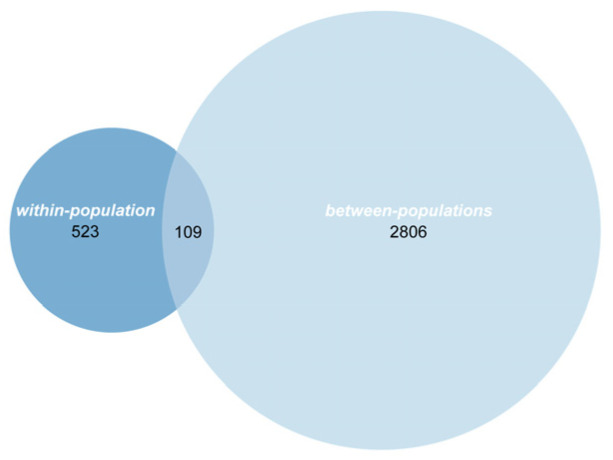
Venn diagram showing the number of genes shared between within-population and between-population analyses.

We compared the genes detected by within-population (e.g., Tajima’s D and iHS) and between-population (e.g., XP-CLR) analyses. A total of 109 genes were shared between the two sets, indicating overlap in selection signals (see Appendix A Figure A1 and Supplementary Table S1).

### 3.5. Gene Set Enrichment Analysis

#### 3.5.1. Gene Set Enrichment Analysis Within-Population

**Figure 5:**
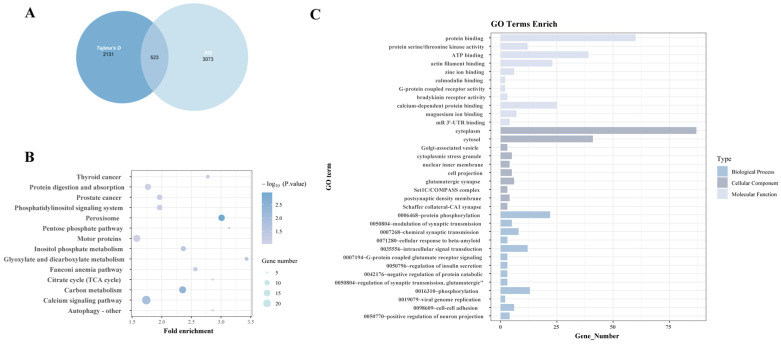
Functional enrichment analysis of genes identified from selection signatures analysis within the population. (**A**) The number of genes identified by the two selection signature methods and their overlap. (**B**) Significant GO enrichment terms for the overlap genes (*p* < 0.05). (**C**) Significant KEGG enrichment terms for the overlap genes (*p* < 0.05).

A total of 2131 and 3073 genes were identified using Tajima’s D and iHS, respectively, among which 523 overlapping genes were subjected to GO and KEGG pathway enrichment analyses (Figure 5).

The pathways related to reproduction in these GO and KEGG pathways include the calcium signaling pathway [26] and zinc ion binding [27]. The domestication-related pathways include glutamatergic synapse [28], modulation of synaptic transmission, and chemical synaptic transmission [29]. Pathways associated with growth include calcium-dependent protein binding [26] and regulation of insulin secretion [30] (Table 2).

#### 3.5.2. Gene Set Enrichment Analysis Between-Populations

**Figure 6:**
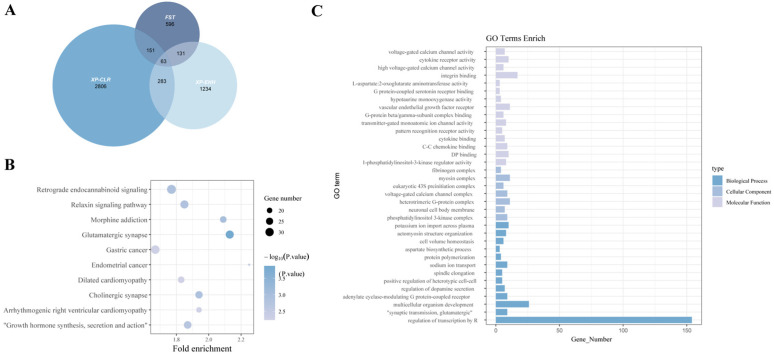
Functional enrichment analysis of genes identified from selection signal analysis between subpopulations. (**A**) The number of genes identified by the three selection signal methods and their overlap. (**B**) Significant GO enrichment terms for the XP-CLR–identified genes (*p* < 0.05). (**C**) Significant KEGG enrichment terms for the XP-CLR-identified genes (*p* < 0.05).

A total of 2806 genes were identified by XP-CLR and used for GO and KEGG enrichment analyses. Although 63 genes were jointly detected by XP-CLR, XP-EHH, and *F_ST_*, only the genes detected by XP-CLR were included in the enrichment analysis (Figure 6).

The pathways related to reproduction in these GO and KEGG pathways include placental growth factor receptor activity [31], Relaxin signaling pathway [32], Endometrial cancer [33] and regulation of dopamine secretion [34]. Pathways associated with growth include insulin receptor activity [35], growth hormone synthesis, secretion and action [36] and Signaling pathways regulating pluripotency of stem cells [37] (Table 3).

## 4. Discussion

PCA of the 41 Duolang sheep revealed no distinct clustering between single- and multi-lamb individuals. This result is unsurprising: principal components primarily summarize broad demographic structure rather than trait-specific variation, and fertility is a polygenic trait whose frequency differences between the two reproductive classes are insufficient to generate distinct clusters. Nevertheless, contrasting the same individuals by phenotype remains appropriate for selection scans: between-group statistics (XP-CLR, *F_ST_*, XP-EHH) can still pinpoint loci with allele-frequency shifts, and within-population metrics (Tajima’s D, iHS) provide complementary evidence. Therefore, the absence of PCA separation does not compromise our combined analytical strategy for identifying candidate fertility genes.

Linkage disequilibrium (LD) decay analysis shows that, compared to other livestock breeds, such as crossbred Vrindavani cattle, with a mean r^2^ of 0.21 at a marker distance of 25–50 kb, and Landrace pigs, with a mean r^2^ of 0.50 at an average distance of 37 kb [38,39], the linkage disequilibrium between non-allelic genes in Duolang sheep decays more rapidly with increasing distance: at a distance of 50 kb, the average r^2^ is less than 0.05. This suggests that Duolang sheep have been subject to relatively low levels of artificial selection, considering the relatively short breeding history.

Tajima’s D selection method compares nucleotide diversity (π) with the number of segregating sites (θ). Specifically, Tajima’s D can be used to detect neutral selection, balancing selection, and purifying selection [4]. iHS is based on the concepts of haplotype extension and linkage disequilibrium, making it particularly suitable for detecting recent positive selection. Positive iHS values indicate a tendency to retain new mutations, negative iHS values indicate a tendency to remove new mutations, and high absolute iHS values indicate strong selection signatures [40]. Furthermore, the iHS approach is particularly effective at detecting partial selective sweeps, while Tajima’s D test is notably powerful for identifying fixation signatures. Therefore, combining these two methods can be effective in identifying signatures of selection in the population. In the selection signal analysis, the two methods identified many genes with a significant overlap, indicating good consistency between the results of the two scanning methods. Enrichment analysis of the intersection of genes detected by Tajima’s D and iHS methods shows that these gene groups have significant functional enrichment in domestication, reproduction, and growth traits. This indicates that the population has undergone sustained selection for these three traits.

XP-CLR (cross-population composite likelihood ratio) was employed as the primary method for detecting selection. XP-CLR evaluates multilocus allele-frequency differentiation and incorporates local linkage disequilibrium. This framework enables the identification of coordinated allele-frequency shifts across linked SNPs. It does not depend on extreme signals at individual loci. These properties make XP-CLR particularly effective for detecting selection between two groups within the same breed. FST and XP-EHH were applied as complementary approaches. *F_ST_* measures genetic differentiation between populations, reflecting the proportion of genetic variation attributed to population structure. High *F_ST_* values indicate significant genetic divergence, often suggesting differential selective pressures across populations [7]. Conversely, XP-EHH evaluates haplotype homozygosity between two populations to detect selective sweeps that have occurred in one population but not in the other [8]. Positive XP-EHH values identify regions with extended haplotypes due to recent positive selection in one population, while the other population shows less extension. Integrating XP-CLR as the primary method with FST and XP-EHH as supporting metrics allows us to capture multilocus differentiation, single-locus divergence, and haplotype-based sweep signatures. This combined strategy provides a robust approach for detecting and characterizing selective signatures.

The results of the gene enrichment analysis based on four selection signatures indicate that the Duolang sheep population has historically undergone selection for traits related to reproduction, domestication, and growth. These traits align with human needs for this livestock species. It is well known that all mammalian species studied so far require calcium oscillations for fertilization. Calcium-mediated signal transduction is considered fundamental to many essential processes involved in mammalian development and reproduction [26]. Prolactin is a hormone essential for normal reproduction, playing a crucial role in various tissues, including the mammary glands and ovarian follicles [41]. Similarly, studies have shown that during mammalian fertilization, zinc is ejected into the extracellular environment in a series of coordinated events known as the zinc spark. Zinc-ion-dependent signaling is crucial for oocyte maturation, fertilization (activation), and subsequent embryo development [27]. The significant enrichment of genes in reproduction-related pathways suggests that reproductive traits have been a major focus in the breeding of Duolang sheep. The domestication, reproduction, and growth of animals are complex phenotypes. Studies have shown that domestication behavior is influenced by various hormones and neurotransmitters [3,42]. Enrichment analysis of the selected genes revealed pathways related to neural signaling and synaptic transmission, such as the glutamatergic synapse, modulation of synaptic transmission, and chemical synaptic transmission [28,30]. Research has also shown that calcium ion binding [43] and insulin [30] play important roles in the growth, development, and metabolism of animals.

Based on the selection signature detection methods, we identified *ESRRA*, *ESRRB*, *OXT*, *FSHR*, *ESR2*, *GNRHR*, and *BMPR1B* as candidate genes. A significant number of loci in these genes showed notable differences in genotype frequencies between single and multiple birth groups. The impact of the *BMPRIB* gene on litter size in sheep has long been a major research area. Numerous studies on sheep have shown that the Bone Morphogenetic Protein Receptor IB (*BMPRIB*) gene is one of the candidate genes for increasing litter size in sheep. Specifically, a variant of *BMPRIB* known as *FecB* is strongly associated with prolificacy traits in sheep [44,45]. *BMPR1B* promotes follicular development and ovarian granulosa cell proliferation, thereby affecting ovulation in mammals [46]. *ESRRA* and *ESRRB* are receptors for estrogen-related receptor alpha and estrogen-related receptor beta, respectively. Previous studies have shown that *ESRRA*, a key regulator of metabolic stress, promotes osteogenesis and vascular formation in adipocyte-rich bone marrow [47], while *ESRRB* often plays a role in mammalian embryos and is related to early embryonic development and germ cell formation [48]. However, there are currently no reports linking *ESRRB* and *ESRRA* to litter size in sheep. Oxytocin (*OXT*), a small peptide hormone mainly synthetized in the magnocellular neurons of the hypothalamus, would facilitate the contractility of the myometrium [49]. The study shows that improvements in fertility, in terms of both farrowing rates and litter size, have been documented in sows inseminated with semen doses supplemented with *OXT* [50]. *OXT* influences various biological functions, including reproduction. Twin ewes with the CC genotype had higher reproductive hormone levels and greater prolificacy [51]. *FSHR* is crucial for the development of gametes in both females and males [52]. *FSH* plays a crucial role in ovarian folliculogenesis and the development of antral follicles, and it works in conjunction with luteinizing hormone (LH) to stimulate the growth of pre-ovulatory follicles. *FSH* influences the proliferation of granulosa cells and the synthesis of estrogen by granulosa cells. Estrogen is essential for the maturation and growth of follicles and prepares them for ovulation triggered by the LH surge [53]. Therefore, the *FSHR* gene should have a strong correlation with litter size traits in sheep. The Gonadotropin-releasing hormone receptor (*GnRHR*) plays a critical role in the hypothalamo–pituitary–gonadal axis by mediating the signal transduction of gonadotropin-releasing hormone, which stimulates the synthesis and release of pituitary gonadotropins (luteinizing and follicle-stimulating hormones), ultimately regulating ovulation and gonadal steroid production [54]. Studies have found that specific polymorphisms in the *GnRHR* gene may affect litter size in West African Dwarf goats [55]. Therefore, *GnRHR* is considered a candidate gene for litter size. Caution is warranted because some subgroups have relatively small sample sizes, and the detected selection signals should therefore be considered putative. Validation in larger populations will be important to confirm their functional relevance.

## 5. Conclusions

This study presents the first genomic landscape of Xinjiang’s Duolang sheep through whole-genome resequencing data from 60 individuals, revealing conserved genetic diversity characterized by low *F*_ROH_ (0.00023), low *F*_HOM_ (0.01520), and rapid LD decay (r^2^ < 0.1 within 10 kb). Subsequently, Tajima’s D, iHS, *F_ST_* and XP-EHH selection signature detection methods were employed, and several candidate genes were screened, including *ESRRA*, *ESRRB*, *OXT*, *FSHR*, *ESR2*, *GNRHR*, and *BMPR1B*. Finally, gene enrichment analysis supported the involvement of the selected genes in selection-related biological processes in the Duolang sheep population. These results provide a genomic foundation for molecular-assisted breeding, particularly for improving prolificacy. Although the findings are based on a limited sample size and should be considered preliminary, they highlight candidate genes that warrant further functional validation. Future studies should also assess genotype–environment interactions in larger populations.

## Figures and Tables

**Table 1:** Candidate genes related to reproduction revealed by selection signatures.

Method	Official Symbol	Official Full Name
Tajima’s D	*ESRRA*	estrogen-related receptor alpha
	*ESRRB*	estrogen-related receptor beta
iHS	*OXT*	oxytocin/neurophysin I prepropeptide
	*FSHR*	follicle-stimulating hormone receptor
	*ESR2*	estrogen receptor 2
	*ESRRB*	estrogen-related receptor beta
	*GNRHR*	gonadotropin-releasing hormone receptor
	*BMPR1B*	bone morphogenetic protein receptor type 1B
XP-CLR	*ESRRA*	estrogen-related receptor alpha
	*BMPR1B*	bone morphogenetic protein receptor type 1B
	*IGF1R*	insulin-like growth factor 1 receptor
XP-EHH	*BMPR1B*	bone morphogenetic protein receptor type 1B

**Table 2:** GO terms and KEGG pathways associated with reproduction, domestication, and growth processes.

	Categories	Term	Count	*p* Value
Reproduction	KEGG_PATHWAY	Calcium signaling pathway	15	0.000
	GOTERM_MF_DIRECT	Zinc ion binding	22	0.032
Domestication	GOTERM_CC_DIRECT	Glutamatergic synapse	6	0.027
	GOTERM_BP_DIRECT	Modulation of synaptic transmission	5	0.002
	GOTERM_BP_DIRECT	Chemical synaptic transmission	8	0.005
Growth	GOTERM_MF_DIRECT	Calcium-dependent protein binding	3	0.037
	GOTERM_BP_DIRECT	Regulation of insulin secretion	3	0.022

**Table 3:** GO terms and KEGG pathways associated with reproduction and growth processes.

	Categories	Term	Count	*p* Value
Reproduction	GOTERM_MF_DIRECT	placental growth factor receptor activity	10	0.05
	GOTERM_BP_DIRECT	regulation of dopamine secretion	7	0.01
	KEGG_PATHWAY	Relaxin signaling pathway	29	0.05
	KEGG_PATHWAY	Endometrial cancer	16	0.00
Growth	GOTERM_MF_DIRECT	insulin receptor activity	10	0.05
	KEGG_PATHWAY	Growth hormone synthesis, secretion, and action	27	0.00
	KEGG_PATHWAY	Signaling pathways regulating pluripotency of stem cells	29	0.00

## Data Availability

The data that support the findings of this study are available from the corresponding author upon reasonable request.

## Supplementary Materials

The following supporting information can be downloaded at https://www.mdpi.com/article/10.3390/ani15233466/s1, Table S1: List of genes identified by all five methods.

## Abbreviations

The following abbreviations are used in this manuscript:

SNP	single nucleotide polymorphism
PCA	principal components analysis
QD	Quality by Depth
MQ	Mapping Quality
SOR	Strand Odds Ratio
MAF	Minor allele frequency
EHH	Extended haplotype homozygosity
iHS	Integrated haplotype score
KEGG	Kyoto encyclopedia of Genes and Genomes
GO	Gene Ontology
CC	Cellular component
MF	Molecular function
BP	Biological process
